# Circulating tumor cells as a prognostic and predictive marker in gastrointestinal stromal tumors: a prospective study

**DOI:** 10.18632/oncotarget.9128

**Published:** 2016-05-02

**Authors:** Qiang Li, Xiaofei Zhi, Jianping Zhou, Ran Tao, Jiaxuan Zhang, Peisheng Chen, Oluf Dimitri Røe, Luning Sun, Lilin Ma

**Affiliations:** ^1^ Department of General Surgery, Affiliated Hospital of Nantong University, Nantong, China; ^2^ Department of Gastrointestinal Surgery, Affiliated Yixing Hospital of Jiangsu University, Yixing, China; ^3^ Clinical Cancer Research Center, Aalborg University Hospital, Clinical Institute, Aalborg, Denmark; ^4^ Cancer Clinic, Levanger Hospital, Nord-Trøndelag Health Trust, Levanger, Norway; ^5^ Department of Cancer Research and Molecular Medicine, Norwegian University of Science and Technology, Trondheim, Norway; ^6^ Research Division of Clinical Pharmacology, First Affiliated Hospital of Nanjing Medical University, Nanjing, China

**Keywords:** circulating tumor cells, ANO1, DOG1, gastrointestinal stromal tumors, recurrence

## Abstract

**Background:**

Circulating tumor cells (CTC) are prognostic and predictive for several cancer types. Only limited data exist regarding prognostic or predictive impact of CTC on gastrointestinal stromal tumor (GIST) patients. The aim of our study was to elucidate the role of CTC in GIST patients.

**Results:**

A total of 121 GIST patients and 54 non-GIST samples were enrolled in the study. The cutoff value for ANO1 positive was 3*10^−5^ and 65 (54%) GIST patients were defined as ANO1 positive. ANO1s were more frequently detected in unresectable patients. Tumor size, mitotic count and risk level were associated with ANO1 detection in resectable GIST patients. The presence of ANO1 significantly correlated with poor disease-free survival (15.3 versus 19.6 months, p = 0.038). Most patients turned ANO1-negative after surgery and inversely, all 21 patients with recurrence turned ANO1-positive with high ANO1 expression levels. Moreover, in the neoadjuvant setting, decline of ANO1 expression level correlated with the response of imatinib.

**Methods:**

Cells from peripheral blood mononuclear cells tested positive for anoctamin 1, calcium activated chloride channel, ANO1 (DOG1) were considered as tumor CTC of GISTs. The expression levels of ANO1 were determined using quantitative real-time polymerase chain reaction (qRT-PCR). The highest level of ANO1 expression in non-GIST samples was used as the “cutoff” value.

**Conclusion:**

ANO1 detection by qRT-PCR in peripheral blood is of clinical potential for monitoring recurrence and evaluating therapeutic efficacy of imatinib for GIST patients.

## INTRODUCTION

Gastrointestinal stromal tumors (GISTs) are the most common mesenchymal tumor of the digestive system with an incidence of about 15 cases per million per year [1]. GISTs are believed to originate from the interstitial Cajal cells (ICC) in normal bowel wall or from precursors of these cells [2]. Recently, anoctamin 1, calcium activated chloride channel (ANO1) previously called DOG1 (Discovered On Gastrointestinal tumor protein 1), was found widely expressed in GIST, even in c-KIT negative tumors, and has been shown as a sensitive and specific immunohistochemical marker for GIST [3, 4].

Most GIST patients are asymptomatic, and often incidentally detected [5–9]. For patients with primary localized GIST, surgery is the standard curative treatment. However, even with complete resection, the rate of recurrence may be as high as 33% in five years [10, 11]. Moreover, with imatinib treatment, 20% of GIST patients experience tumor growth within first 6 months [12]. However, except imaging, there are no reliable biological tools to follow the disease status over time.

In recent years, circulating tumor cells (CTCs) detected non-invasively in “liquid biopsies” have been widely discussed in the field of monitoring cancer [13]. In several cancer types, such as breast, prostate, colon and lung cancer, CTCs has shown a significant correlation with clinical outcome [14–18]. Technological advances have enabled further study of the CTC as a prognostic and predictive marker [19]. However, the characteristics of CTCs in patients with GIST remain unknown.

In the current study, we investigated the feasibility of detecting ANO1 based on its expression in peripheral blood of patients with GIST and determine the correlation between the presence of ANO1 and clinical outcome of GIST.

## RESULTS

### Clinicopathological characteristics

**Figure 1:**
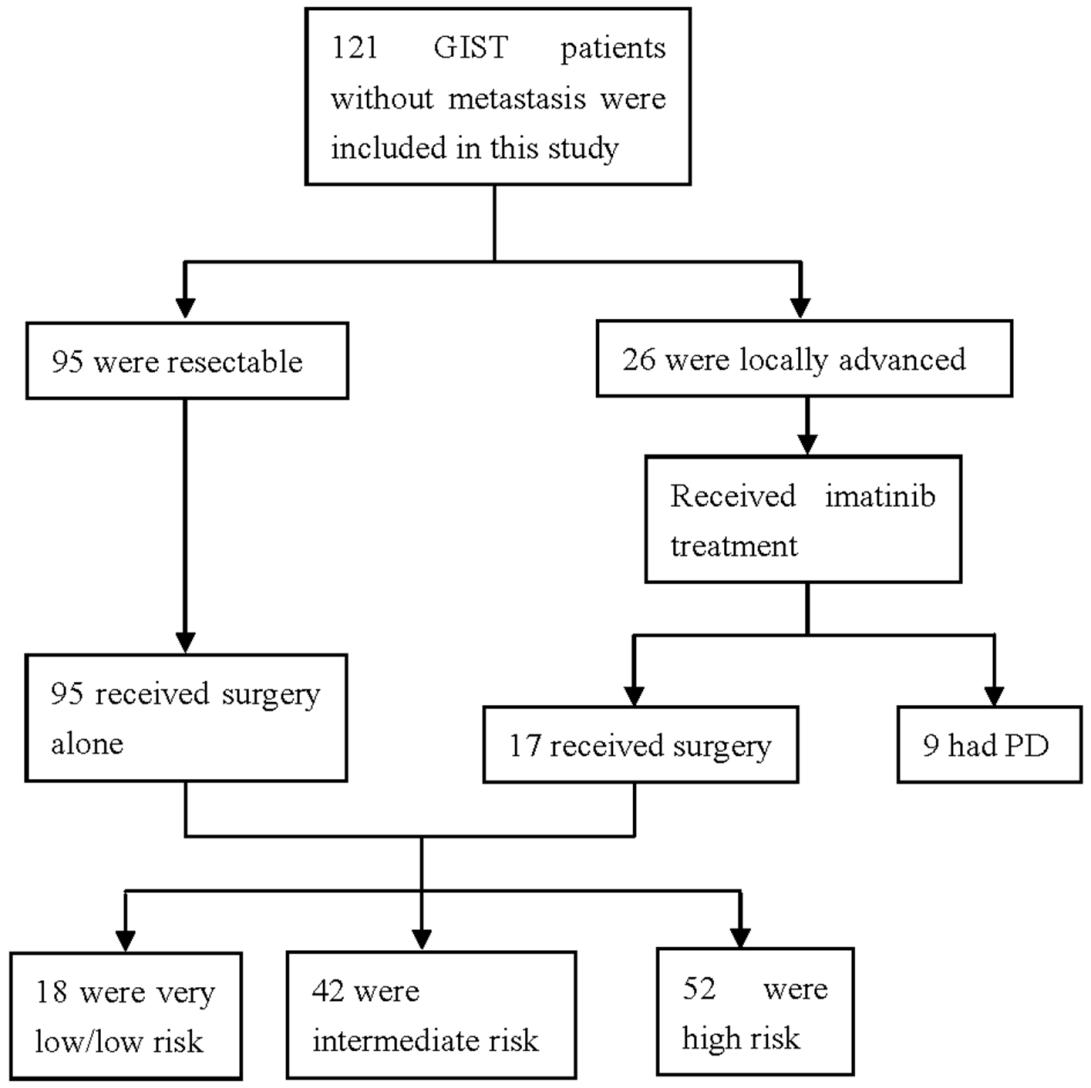
Flowchart of the study

A total of 121 GIST patients were included. All GISTs were determined as ANO1-postive. Of these patients, 26 received neoadjuvant imatinib for 6-12 months before surgery according to National Comprehensive Cancer Network Guideline (NCCN) [20]. Seventeen of 26 GIST patients had R0 resection after imatinib, while 9 had progressive disease. A total of 112 patients including 68 men and 44 women received surgery. Of these, 46 cases had disease located in the stomach (41.1%), 54 cases located in the small intestine (48%), 4 cases in the colorectum (3.6%), 6 cases in the abdominal cavity (5.4%), and 2 cases in the mesenterium (1.8%). According to Fletcher risk classification, 52 of these 112 GIST patients were characterized as high risk (46.4%), 42 as intermediate risk (37.5%), and 18 as low or very low risk (16.1%). The demographic and clinical features of patients are summarized in Table 1. Patients with intermediate or high risk received adjuvant therapy with imatinib according to the guideline of NCCN [20]. Study flowchart is shown in Figure 1.

**Table 1:** Association between resectable GIST patients' clinicopathological characteristics and CTC status

Variable	Overall (n = 112)	CTC negative (n = 54)		CTC positive (n = 58)		P-value
		N	%	N	%	
Gender						
Male	68	36	32.14	32	28.57	0.248
Female	44	18	16.07	26	23.21	
Tumor location						
Stomach	46	24	21.43	22	19.64	0.611
Small intestine	54	26	23.21	28	25.00	
Colorectum	4	2	1.78	2	1.78	
Abdominal cavity	6	1	0.89	5	4.46	
Mesenteriolum	2	1	0.89	1	0.89	
Tumor size						
≤ 5 cm	44	32	28.57	12	10.71	0.000
≤ 10 m	48	17	15.18	31	27.68	
> 10 cm	20	5	4.46	15	13.39	
Mitotic count						
≤ 5 /50HPF	65	39	34.82	26	23.21	0.011
≤ 10 /50HPF	37	11	9.82	26	23.21	
> 10 /50HPF	10	4	3.57	6	5.36	
Risk level						
Very low/low	18	13	11.61	5	4.46	0.026
Intermediate	42	22	19.64	20	17.86	
High	52	19	16.96	33	29.46	
Morphology						
Spindle	92	42	37.5	50	44.64	0.508
Epithelioid	5	3	2.68	2	1.79	
Mixed	15	9	8.03	6	5.36	
Ki-67						
≤ 5%	60	33	29.46	27	24.11	0.133
≤ 10%	28	9	8.04	19	16.96	
> 10%	24	12	10.71	12	10.71	

### ANO1 is a specific marker of CTC in GIST

**Figure 2:**
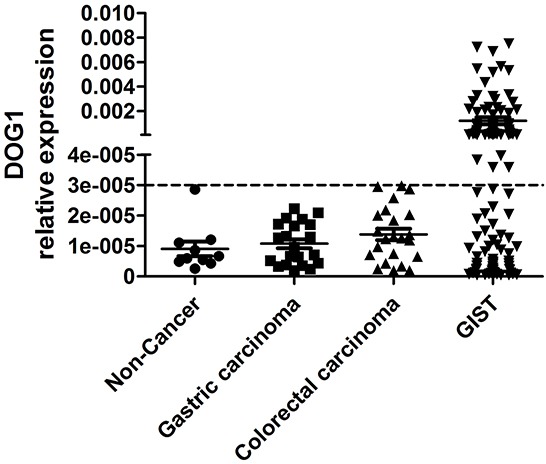
The expression levels of ANO1 in non-cancer healthy donors, in gastric carcinoma patients, in colorectal carcinoma patients and in GIST patients

To analyze the expression level of ANO1 in PBMC from GIST patients, we established the range of expression levels of ANO1 in non-cancer healthy donors, gastric carcinoma patients and colorectal carcinoma patients. The levels of ANO1 transcripts in different samples were calculated relative to that of the housekeeping gene beta-actin. The highest expression levels of ANO1 transcripts relative to beta-actin were 3*10^−5^, 2.2*10^−5^ and 3*10^−5^ in 10 non-cancer healthy donors, 21 gastric carcinoma patients and 23 colorectal carcinoma patients, respectively (Figure 2). Thus, the value of 3*10^−5^ was used as “cutoff” value to determine if GIST patients have ANO1 in the PBMC samples. In our study, 65 GIST patients were defined as ANO1 positive.

### High ANO1 correlated with high risk, large tumor size and high mitotic count

**Figure 3:**
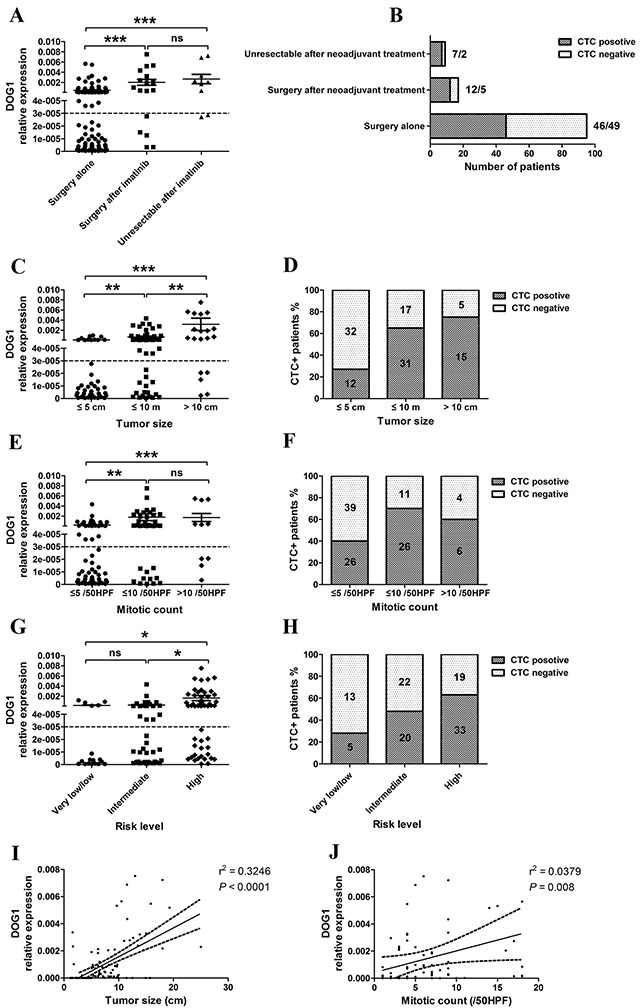
**A, B.** Pre-operative detection of ANO1 expression in GIST patients with imatinib treatment; **C, D.** Correlation between ANO1 expression and tumor size; **E, F.** Correlation between ANO1 expression and mitotic count; **G, H.** Correlation between ANO1 expression and risk levels; **I.** Linear regression between ANO1 expression and tumor size. **J.** Linear regression between ANO1 expression and mitotic count.

In the analysis of preoperative blood samples, 65 (54%) of 121 GIST patients were ANO1 positive, including 26 locally advanced GIST patients who received imatinib treatment before surgery (Figure 3A and 3B). The expression levels of ANO1 in PBMC from locally advanced GIST patients were significantly increased and the positive rate of ANO1 was significantly higher than the patients with resectable GISTs (73.1% versus 54%, p<0.001). Expression levels of ANO1 were significantly associated with tumor size, mitotic count and risk levels. The expression levels of ANO1 and the positive rates of ANO1 were significantly higher in patients with large tumor size, high mitotic count and high risk (Figure 3C-3H). Linear regression also confirmed the significant correlation (r^2^=0.3246, p<0.0001) between ANO1 expression and tumor size (Figure 3E), and the significant correlation (r^2^=0.0379, p=0.008) between ANO1 expression and mitotic count (Figure 3F). There was no association between ANO1 and gender, tumor location, morphology or Ki-67.

### Prognostic role of ANO1 in GIST

**Figure 4:**
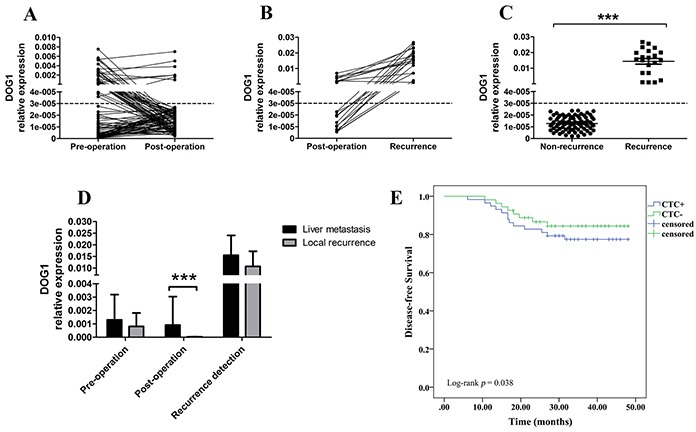
**A.** ANO1 expression trend of GIST patients pre-operation and post-operation. **B.** ANO1 expression trend of GIST patients with recurrence. **C.** The expression of ANO1 in non-recurrence and recurrence GIST patients. **D.** The expression of ANO1 at different stages in GIST patients with tumor recurrence. **E.** Kaplan–Meier analysis for disease-free survival according to the preoperative presence (+) or absence (−) of ANO1.

For 112 patients with surgery, we tested ANO1 status before surgery and four weeks after surgery. There were 58 (51.8%) patients with positive ANO1 preoperatively where only seven remained ANO1 positive postoperatively (Figure 4A).

The mean follow-up time was 38 (0-50) months. During the follow-up period, 21 (18.8%) of 112 GIST patients had recurrence after surgery, including 16 (76.2%) in liver and 5 (23.8%) in peritoneal cavity. The median time of recurrence was 17.6 (6.4-47.6) months. Furthermore, the seven patients with consistently positive ANO1 had liver metastasis after surgery (Figure 4B). All the 21 patients with recurrence were, or became ANO1-positive (Figure 4B, Table 2). The expression levels of ANO1 in patients with recurrence were significantly higher than that in patients without recurrence (Figure 4C). In addition, the postoperative expression levels of ANO1 in liver metastatic GIST patients were significantly higher than that in peritoneal cavity (Figure 4D).

**Table 2:** The presence of CTC at different stages in patients with tumor recurence

Site of recurrence	No.	Pre-operation		P-value	Post-operation		P-value	Recurrence detection	
		CTC+	CTC-		CTC+	CTC-		CTC+	CTC-
Liver	16	11	5	0.325	7	9	0.123	16	0
Peritoneal cavity	5	2	3		0	5		5	0

No patient died during the follow-up. The disease free probability at 50 months for GIST patients with positive ANO1 was 77.6% and for those without ANO1 was 86.2%. The presence of ANO1 predicted a significant poor disease free survival (15.3 versus 19.6 months, p = 0.038) (Figure 4E). moreover, multivariate Cox regression analysis indicate that ANO1 copy number in PBMC was an independent positive prognostic factor for GIST patients (Table 3).

**Table 3:** Multivariate Cox regression analysis of CTC and clinicopathologic variables predicting DFS of GIST patients with surgery

Variables	Disease-free survival	
	HR (95% CI)	P-value
Age, years (≤60 vs >60)	0.632 (0.321-1.115)	0.098
Gender (male vs female)	1.257 (0.449-2.793)	0.363
Primary tumor size (stomach vs non-stomach)	1.389 (0.743-3.529)	0.183
Cell type (spindle vs non-spindle)	0.852 (0.267-2.731)	0.512
Risk level (intermediate/high vs very low/low)	1.826 (1.362-2.583)	<0.001
Ki-67 (≤ 5% vs >5%)	0.378 (0.153-1.295)	0.128
CTC (positive vs negative)	1.183 (1.075-2.363)	0.046

### Predictive role of ANO1 for the response rate of neo-adjuvant imatinib

**Figure 5:**
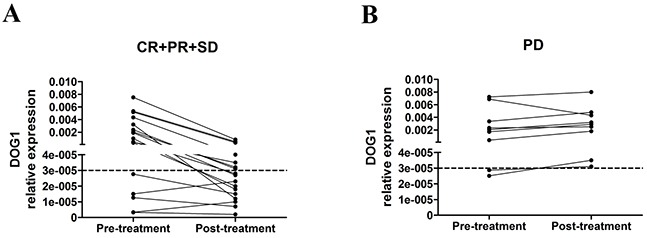
**A.** ANO1 expression trend between pre and post imatinib treatment in partial response (PR) plus stable disease (SD) GIST patients. **B.** ANO1 expression trend between pre- and post-imatinib treatment in progressive disease (PD) GIST patients.

We evaluated the efficacy of imatinib treatment according to the Response Evaluation Criteria in Solid Tumors (RECIST) after three months of neoadjuvant treatment. Of the 26 GIST patients who needed imatinib preoperatively, no patient had complete response, seven had partial response (PR, 26.9%), 10 had stable disease (SD, 38.5%) and 9 had progressive disease (PD, 34.6%). The DCR (CR+PR+SD) was 65.4%. We tested the expression of ANO1 in PBMC pre and post imatinib treatment (Figure 5). The 17 patients with disease control (PR+SD) showed a downward trend of ANO1-expression levels and 10 patients became ANO1 negative. The expression levels of ANO1 in 9 PD patients remained unchanged. These results confirmed that the examination of the copy number of ANO1 in PBMC provides a potential approach for predicting the efficacy of imatinib treatment.

## DISCUSSION

In the present study, we show for the first time that ANO1 copy number in PBMC is a strong prognostic factor for disease-free survival and predictive for therapeutic efficacy of imatinib in GIST patients.

Recently, CTC detection has become an important field of study in biomedical research and has emerged as an early marker of tumor recurrence occurring before clinical symptoms present in various types of tumor [13, 21–23]. However, research on CTC in GIST patients is scarce. Originating from mesenchymal cells, GISTs express unique molecules, where c-KIT and ANO1 have been proven to be key biomarkers. c-KIT (also know as CD117) is a type III receptor tyrosine kinase that plays important roles in hematopoiesis, melanogenesis, and gametogenesis by binding its ligand, stem cell factor (SCF) [24, 25]. Given that there are circulating c-KIT-positive normal cells, including hematopoietic stem cells, it can not be used for GIST CTC dection [24]. The ANO1, a calcium-activated chloride channel that mediates receptor-activated chloride currents in diverse physiologic processes is rarely found overexpressed in other mesenchymal, but also non-mesencymal tumors [26, 27]. Most studies about ANO1 focus on the cancer derived from epidermal tissue, in which ANO1 is non-specific [13, 28–31]. However, GISTs originate from mensenchymal tissue, and ANO1 is a highly sensitive and specific marker for GISTs. Thus, we deduced that CTC detection in PBMC by quantifying ANO1 could be a viable path.

Here, we showed for the first time that the copy number expression of ANO1 in PBMC in GIST patients was significantly higher than that in non-GIST patients. Importantly significantly higher levels were detected in unresectable patients, large tumor size, mitotic count, risk level and poor disease-free survival. Since ANO1 levels dropped after GIST surgery and increased at recurrence, it seems likely that this marker can be used for monitoring after surgery and subclinical detection of recurrence.

Targeted therapies have improved the treatment and survival of cancer patients over the past decade [31, 32]. Imatinib mesylate is the standard first-line therapy of unresectable or metastatic GIST [33]. Currently there is no non-invasive test to monitor tumor response or progression. Also the optimal time point to perform surgery in this setting is also diffuse since we don't know when the maximum effect of neo-adjuvant treatment is. Here, we observed a decline of ANO1 levels in patients that received neo-adjuvant imatinib treatment, while they increased in progressing patients. These results indicate that ANO1 detection could serve as a supplementary approach to evaluate the efficacy of imatinib treatment in GIST patients, as well as using the ANO1 nadir to help define optimal timing of surgery.

This study has some limitations. The number of participants could have been higher, but still, with such a relatively rare disease the number is not insignificant. The qRT-PCR does not confer visualization of ANO1, so one can argue that tumor cells have just been detected indirectly. However, even visualization by e.g. immunohistochemistry would give semi-quantitative information, while qRT-PCR is a quantitative method.

In summary, these data suggest CTC detection by ANO1 as a potentially useful prognostic and predictive biomarker in GIST patients that may help to further stratify risk status within different stages of disease and to monitor the recurrence and metastasis. Extended studies regarding the characteristics of ANO1 in GIST patients are needed to establish this as a clinical method.

## MATERIALS AND METHODS

### Patients

A total of 121 GIST patients were enrolled in our study in Affiliated Hospital of Nantong University and First Affiliated Hospital of Nanjing Medical University from 2011 to 2015. In addition, 10 healthy volunteers, 21 gastric cancer patients and 23 colorectal cancer patients were also included. All the resectable patients had a pathological diagnosis of GIST following surgical resection that met histological or cytological criteria. According to the Response Evaluation Criteria in Solid Tumors (RECIST) of the World Health Organization (WHO) imatinib response evaluation was performed with computed tomography (CT) scan. Response assessment was categorized as complete response (CR), partial response (PR), progressive disease (PD) and stable disease (SD). Informed written consents were obtained by all patients and the study was approved by Affiliated Hospital of Nantong University and First Affiliated Hospital of Nanjing Medical University ethics committees. Adjuvant treatment was done according to current treatment guidelines after obtaining interdisciplinary consensus for each patient. Reporting of the present study was in accordance with the REMARK guidelines [34].

### Extraction of mononuclear cells from peripheral blood

Nearly 10 ml peripheral blood was collected in EDTA vacuum tubes after discarding the first 2 ml of blood to avoid contamination of the blood sample with epithelial cells of skin. Peripheral blood mononuclear cells (PBMC) were isolated by density gradient centrifugation using Lymphocyte Separation Medium (Tianjin, China). The mononuclear cells were washed twice with RPMI Medium 1640 (1x) (Invitrogen, Carlsbad, CA), centrifuged at 1500 rpm for 8 min, then stored at −80°C until needed.

### Extraction of RNA from PBMC and synthesis of cANO1

RNA was extracted from isolated PBMC using TRIZOL reagent (Invitrogen, Carlsbad, CA) according to manufacturer's instructions. After quantification, RNA was used for cANO1 synthesis with a RevertAid First Strand cANO1 Synthesis Kit (Shanghai, China) according to the protocol. The 20μl reaction mixture was incubated at 42°C for 60 minutes and then heated to 72°C for 5 minutes to inactivate the reverse transcriptase, and the mixture was stored at −20°C.

### Quantitative real time-polymerase chain reaction

Quantitative Real Time-Polymerase Chain Reaction (qRT-PCR) was performed using FastStart Universal SYBR Green Master (Rox) (Roche Diagnostics GmbH, Mannheim, Germany). According to the protocol, 20 μl reaction volumes were run containing 2 μl cANO1. The qRT-PCR experiments were performed in 96-well plates in an ABI Prism 7500 (ABI, California, USA). Cycling parameters were as follows: hot start at 95°C for 10 min, 45 cycles of amplification, quantification at 95°C for 15s, 58°C for 1 min, during which time fluorescence was measured, and 72°C for 30 s. Melting curve analysis was performed using continuous fluorescence acquisition from 65°C to 97°C. These cycling parameters generated single amplification for primer set used according to the presence of a single melt peak. β-actin was selected as the internal reference. Each sample was processed in triplicate. Primer sequences which were designed on the basis of the published human gene sequences being as follows: ANO1 (sense: 5′-AGCCACC TCTTCGACAAC-3′, anti-sense: 5′-GACAGCCTCCTC TTCCTCT-3′) and β-actin (sense: 5′-TACTTGCGCTC AGGAGGAGCAA-3′, anti-sense: 5′-GTCCTGTGGCAT CCACGAAACT-3′). Gene expression levels were calculated according to the following equation: 2^−ΔCt^ [ΔCt=Ct (target)- Ct (beta-actin)].

### Statistical methods

Statistical analysis was conducted using the SPSS17.0 statistical software (SPSS, Chicago, USA). Data are presented as mean ± SD. The association of ANO1 detection with clinicopathological variables was evaluated using the Chi-square test. Survival curves were constructed according to the Kaplan–Meier method and compared using the log-rank test. A P-value < 0.05 was considered to indicate a statistically significant difference.
